# A Machine Learning App for Monitoring Physical Therapy at Home

**DOI:** 10.3390/s24010158

**Published:** 2023-12-27

**Authors:** Bruno Pereira, Bruno Cunha, Paula Viana, Maria Lopes, Ana S. C. Melo, Andreia S. P. Sousa

**Affiliations:** 1 Instituto Superior de Engenharia do Porto (ISEP), Polytechnic of Porto, Rua Dr. António Bernardino de Almeida, 4249-015 Porto, Portugal; 1201441@isep.ipp.pt (B.P.); pmv@isep.ipp.pt (P.V.); 2 Institute for Systems and Computer Engineering, Technology and Science (INESC TEC), 4200-465 Porto, Portugal; 3 Center for Rehabilitation Research, Human Movement System (Re)habilitation Area, School of Health, Polytechnic of Porto, Rua Dr. António Bernardino de Almeida, 400, 4200-072 Porto, Portugal; mariavieiralopes@hotmail.com (M.L.); asp@ess.ipp.pt (A.S.P.S.); 4 Porto Biomechanics Laboratory (LABIOMEP-UP), University of Porto, Rua Dr. Plácido Costa, 91, 4200-450 Porto, Portugal; 5 Center for Interdisciplinary Applied Research in Health (CIIAS), School of Health, Setubal Polytechnic Institute, Campus do IPS Estefanilha, 2914-503 Setubal, Portugal; 6 Research Centre in Physical Activity, Health and Leisure (CIAFEL), Faculty of Sport, University of Porto, Rua Dr. Plácido Costa, 91, 4200-450 Porto, Portugal

**Keywords:** pose estimation, exercise evaluation, mobile health, remote monitoring, rehabilitation

## Abstract

Shoulder rehabilitation is a process that requires physical therapy sessions to recover the mobility of the affected limbs. However, these sessions are often limited by the availability and cost of specialized technicians, as well as the patient’s travel to the session locations. This paper presents a novel smartphone-based approach using a pose estimation algorithm to evaluate the quality of the movements and provide feedback, allowing patients to perform autonomous recovery sessions. This paper reviews the state of the art in wearable devices and camera-based systems for human body detection and rehabilitation support and describes the system developed, which uses MediaPipe to extract the coordinates of 33 key points on the patient’s body and compares them with reference videos made by professional physiotherapists using cosine similarity and dynamic time warping. This paper also presents a clinical study that uses QTM, an optoelectronic system for motion capture, to validate the methods used by the smartphone application. The results show that there are statistically significant differences between the three methods for different exercises, highlighting the importance of selecting an appropriate method for specific exercises. This paper discusses the implications and limitations of the findings and suggests directions for future research.

## 1. Introduction

Accidents in our day-to-day activities are inevitable and can cause injuries that require a rehabilitation process. In order to recover some or all of the mobility affected by these accidents, the majority of solutions require physiotherapy sessions.

In a rehabilitation scenario supported by remote physiotherapy sessions, the target patient faces great difficulties in correctly executing the proposed exercises autonomously. In addition, physiotherapists do not have the tools to correctly monitor these homemade exercises, assess the degree of perfection with which they are performed, or their real relevance to recovery.

In such a scenario, the use of devices and tools to support and monitor these autonomous sessions can have a significant impact on these processes, helping to improve the effectiveness of treatments. The fact that recent years have seen an evolution in the processing capacity of mobile devices, which has also been accompanied by an evolution in the performance of data processing algorithms, particularly in the area of computer vision, makes these types of devices strong candidates for implementing solutions in the area of health and well-being.

Although wearable devices and camera-based systems have been used for human body detection and rehabilitation support, these solutions often require expensive hardware or lack the ability to provide real-time feedback to patients. This represents a significant gap in the literature, as affordable and effective rehabilitation solutions are crucial for patient recovery. Our study addresses this gap by introducing a smartphone-based approach for shoulder rehabilitation. By leveraging a pose estimation algorithm, our system can evaluate the quality of patients’ movements and provide immediate feedback, all using a device that most people already own. This not only makes our solution more accessible but also allows for more consistent and effective rehabilitation sessions.

This project aimed to develop a tool that can help both therapists and patients, reducing the degree of uncertainty imposed by autonomous exercises. To this end, a mobile application was created to collect information about the execution of autonomous exercises. The app uses pose estimation mechanisms and provides the patient with feedback on the quality of their execution of the exercises.

The work for this manuscript builds upon some of our previous work [1], where we detailed how to validate a smartphone application that, through video recording monitors, supervises the execution of therapeutic shoulder exercises and gives the user feedback regarding the movement quality.

The remaining sections of this paper are organized as follows. Section 2 presents a survey of current state-of-the-art academic and commercialized applications in human body detection and rehabilitation support and discusses their weaknesses and strengths. Section 3 presents the application developed, describing its main functionalities and the algorithm used for automatically inferring the quality of the exercises executed. Section 4 describes the testbed implemented for validating our solution. The results are presented in Section 5 and discussed in Section 6. Finally, Section 7 summarizes the main conclusions and suggests some future paths.

## 2. Related Works

Numerous commercial and academic projects have developed ways of tracking the human body, using these data for various purposes, such as counting steps, measuring heartbeats, or detecting body movements. Most of these projects use the human body’s detection capabilities to help with physical exercises; however, there are no major solutions on the market dedicated to rehabilitation programs.

### 2.1. Wearable Device-Based Approaches

Wearable devices play an important role in rehabilitation processes, especially for patients with neurological or locomotive disabilities. These devices have sensors capable of collecting and analyzing kinematic data that can be used to evaluate the motor skills of patients, making the process of monitoring the rehabilitation stages easier [2]. Several solutions based on portable devices have been used for either rehabilitation or gymnastic purposes.

GymApp [3] is an application designed with workout tracking in mind. It relies on the inertial sensors of an Android smartwatch and, using a set of pattern recognition algorithms, detects the rate of success in the execution of a planned workout. This application can run solo and does not need the support of any other external device as all the algorithms run on the wearable device. One of the most important aspects of GymApp is its capability to efficiently align two temporal sequences that may vary in speed using the dynamic time warping (DTW) algorithm [4]. This allows for a comparison of two exercises that have been performed at different speeds. The main drawback of this solution in a rehabilitation scenario is that it requires using a number of these devices to guarantee the accurate detection of several key points on the human body in order to increase the confidence of the values collected. In addition, the immediate consequence is the considerable cost for the patient.

Given the diversity of available devices, Bowman et al. [5] carried out a study on portable devices for biofeedback, aiming at identifying the most commonly used sensors and components for different pathologies. To explore these aspects and estimate the effectiveness of biofeedback rehabilitation using portable devices, they conducted a systematic review that can be used as background information for other researchers in the field.

Maceira-Elvira et al. [6] also conducted a review, carried out at the Center for Neuroprosthetics (CNP) and the Brain Mind Institute (BMI) in Switzerland, on the use of wearable sensors in stroke rehabilitation processes, with a particular focus on the upper extremities. A deep analysis of the reliability of the solutions was provided, together with guidelines concerning data acquisition and processing that should be implemented in future works.

The solutions described above have several drawbacks, often pointed out by therapists and patients, which usually lead to this type of approach being avoided. The most relevant drawbacks are the costs associated with the wearable devices, which need to be borne by the patient or the rehabilitation organization, and the time needed to configure and install the sensors during sessions. A question often raised is whether this technology can really improve the workloads of physiotherapists or whether it further increases consultation times and the qualifications required from the therapists.

### 2.2. Camera-Based Approaches

#### 2.2.1. Non-Skeleton-Based

Computer vision in the area of virtual rehabilitation was considered long before skeleton tracking was feasible. Research in this field often uses indirect methods of detecting the position of the human body, such as color or object detection. Sucar et al. [7] used skin color to detect the hands and thus assist in therapy movements. This system required the use of a green ball attached to a hand gripper to facilitate detection. Patients were then asked to move their arms in a simulated environment. Although the system was only tested in a controlled environment, it showed promising results.

Nevertheless, solutions that do not use skeleton detection are immensely limited due to their inability to detect joints; such methods are only capable of detecting the movement of a specific part of the body, such as the arms [7,8]. Ways of compensating for this limitation, but not completely overcoming it, include the use of other auxiliary computer vision methods such as silhouette detection [8,9,10]. The project led by Natarajan et al. [10] used depth information to discriminate the background of the object under analysis. This method, aided by morphological selection operations, made it possible to select the human silhouette, thereby enhancing accuracy.

#### 2.2.2. Skeleton-Based

With the introduction of Microsoft Kinect in 2010, solutions based on skeleton detection have become increasingly possible. Systems such as the ones proposed by Chang et al. [11], Fern et al. [12], and Da Gama et al. [13] use Kinect to accurately detect patient movements. These systems can detect and extract key points on the human body, whose coordinates are then used to form vectors and identify angles between different body parts. In order to reduce complexity, the number of key points used is reduced to the smallest value required for evaluation. However, this simplification introduces some limitations, as the use of deep learning, which can help increase accuracy, is hindered by the small size of the datasets.

Instant feedback, with the aim of providing real-time instructions and correcting the exercise while it is being performed, has also been considered by researchers. Ghali et al. [14] simultaneously implemented object and event detection techniques to determine the position of the human body. They then compared the real-time information with previously recorded videos to verify the accuracy of the detection. MirrARbilitation [15] uses Kinect’s RGB camera to capture exercises and detect human poses. The system is capable of detecting up to 20 key points on the human body. Therapists have the flexibility to adjust or modify the proposed exercises and change the error tolerance in the precision of the movement or the time taken to perform it. The system uses angles to evaluate the current state of the exercise, offering real-time suggestions and adjustments. The system proposed by Su et al. [16] follows the same approach using a Kinect-only implementation, offering individual feedback about each limb of the patient.

However, these systems are still quite complex, rely on the Kinect sensor as an external device, and require considerable computing power, which increases the cost of implementing the given solution.

#### 2.2.3. Virtual Reality-Based

With the evolution and integration of virtual reality systems into society, including in multimedia and the medical industry, some systems have integrated this technology as a basis for aiding rehabilitation. The work of Adams et al. [17] and the VRehab [18] system are examples of the use of the Kinect sensor to detect a patient’s movements. Both solutions have shown satisfactory results, aligning closely with performance evaluations conducted by therapists in the field [8]. Despite the excellent results, it is worth considering the costs associated with this type of solution. Besides requiring the Kinect sensor, it is also necessary to purchase virtual reality equipment.

### 2.3. Other Worthy Applications

RehabGuru [19] and PT Pal Pro [20] are Android and IOS applications that offer a great variety of rehabilitation exercises. They include short videos and animations that help the patient understand the exercise that he/she is expected to do. However, they do not utilize any body detection mechanisms and, therefore, do not offer any feedback.

Korczak et al. [21] analyzed how mobile applications are used and how effective they are in monitoring the physical activity, rehabilitation, and education of people with intellectual and/or other disabilities. From a panoply of 115 scientific articles related to the topic, 23 were thoroughly analyzed. The authors concluded that interest in using mobile applications for working with people with intellectual or physical disabilities has increased significantly since 2015 and that the majority of therapists rely on mobile applications to support the rehabilitation processes of people with disabilities. Also, several home exercise platforms have gained popularity [22]. These usually offer live and on-demand classes, are updated daily, and can be used for activities such as strength training, cycling, or dancing. These platforms provide convenience, variety, progress tracking, and tips for improvements or goal setting [22]. We believe that these conclusions are encouraging for the use of standard mobile devices in these scenarios.

### 2.4. Similarity Metrics in Motion Capture Data

For the analysis of motion capture data, similarity metrics are crucial for comparing and classifying different motion patterns. This section gives an overview of the two similarity metrics employed in this work: cosine similarity and DTW.

#### 2.4.1. Cosine Similarity

Cosine similarity is a measure of similarity between two non-zero vectors within an inner-product space. It is calculated as the cosine of the angle between the vectors, which is equivalent to the inner product of these same vectors normalized to both have a length of 1.

In the context of motion capture data, cosine similarity can be used to compare vectors representing different motion patterns. For instance, if two vectors represent the motion patterns of two different exercises, the cosine similarity between these vectors can be calculated to quantify the similarity of these exercises. The closer the cosine similarity is to 1, the more similar the exercises.

A practical use case of cosine similarity in motion capture data is in the field of multimedia applications. In the paper by Sedmidubsky et al. [23], each short motion is encoded into a compact visual representation from which a highly descriptive 4096-dimensional feature vector is extracted using a fine-tuned deep convolutional neural network. The fixed-size features are compared using the Euclidean distance, which enables efficient motion indexing by any metric-based index structure. The goal is to make this approach more tolerant to variations in movement speed and/or lower data quality.

#### 2.4.2. Dynamic Time Warping

DTW is a method that allows a flexible comparison of two temporal sequences that may vary in speed and timing. It has been widely used in various fields, including speech recognition, data mining, and notably, motion capture, where it can effectively measure the similarity between different motion patterns. A comprehensive study by Switonski et al. [24] on the classification of motion capture data based on DTW presented both the theoretical descriptions of all applied and newly proposed methods and the experimentally obtained results on a real dataset of human gait.

By employing these two methods, this work aims to provide a robust and flexible approach to the analysis of motion capture data. The following sections delve into the application and results of these methods in the context of the proposed app for physical therapy.

## 3. Proposed Solution

Our work aims to overcome the shortcomings identified in former works by developing an app-based solution capable of detecting human poses and automatically analyzing the quality of the exercises performed by the patient, facilitating feedback on the required corrections. The proposed solution does not require the use of any external sensors, relying solely on the camera and processing power of a generic mobile phone. Additionally, it enables personalizing the exercises, keeping a record of the sessions, and changing the device used while synchronizing the data. Several other functionalities were considered to enable the application to be used universally.

### 3.1. General Functionalities

**Figure 1:**
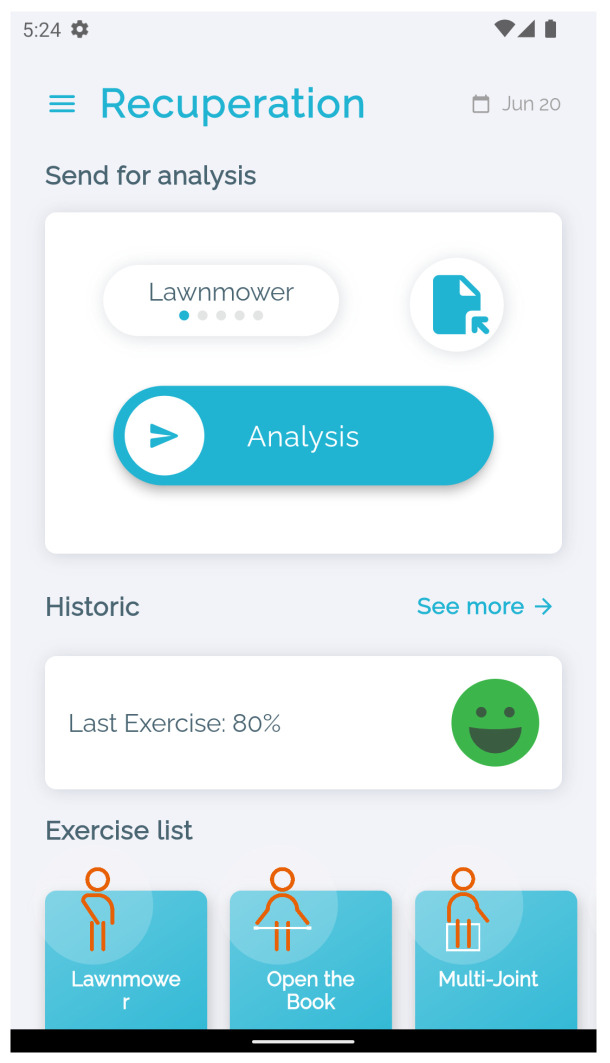
System menu.

The application includes a list of available exercises that the user can choose from. Each exercise is represented in the list by a widget with its name and a small illustration of the exercise (Figure 1). This list can be personalized to include additional ones that are built specifically for a given patient.

**Figure 2:**
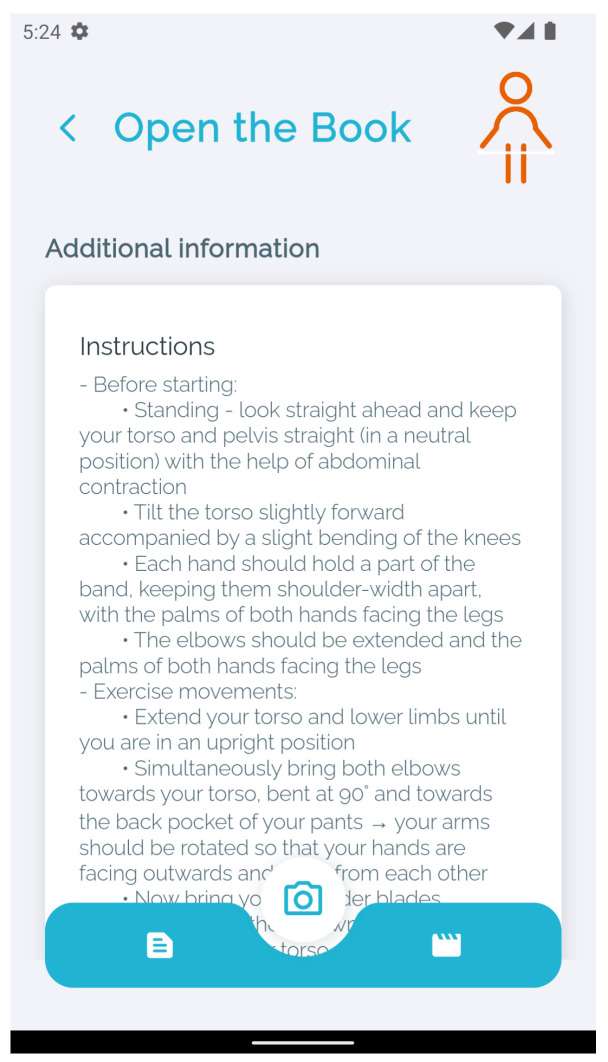
Exercise details.

For each of the exercises, detailed instructions on all the steps are provided, including the time required to perform it and a demonstration video made by professional physiotherapists, where they demonstrate all the stages of the exercise step by step (Figure 2).

The user is also provided with the rate of success of his/her previous sessions. This information is also shared with the physiotherapist so that he/she can follow the evolution of his/her patient. This functionality is based on a scalable and flexible NoSQL cloud database [25] that stores and synchronizes the data. In cases where there is no Internet connection, a temporary local registry is created. After reestablishing a connection, synchronization is performed. The database stores all the information about the session, including the date, type of exercise performed, and rate of success. The full history of exercises can also be accessed from the main menu.

In order to securely save user data in the cloud and enable the same personalized experience across all the user’s devices, a cross-platform authentication service based on the Firebase Authentication framework [25] was implemented, enabling login via Google and Facebook.

The application includes also accessibility options for visually impaired users: a high-contrast GUI, a zoom system, specialized graphic notations, and an audio-based help system supported by the Talkback function of the smartphone. The app is available in three languages: Portuguese, English, and Spanish.

The high-contrast system allows the user to change the application to a black-and-white version, eliminating any distortion introduced by colors that are similar to the background colors. The zoom mode, as the name implies, is a function that allows the user to zoom in on any area of the GUI, allowing people with focal difficulties to read the text without any problems. According to Color Blindness Awareness [26], color blindness affects approximately 1 in 12 men and 1 in 200 women, representing 8.3% of the male population and 0.5% of the female population. In an effort to combat this problem, the application features the ColorAdd [27] system, a unique and inclusive graphic system that allows color-blind people to identify colors through symbols. By associating colors with primary color symbols and combining these symbols to create the remaining colors, a wide variety of different colors can be created. It is even possible to differentiate light colors from dark colors. All these functions can be activated or deactivated in the application settings according to the user’s needs.

For users with complete vision loss, TalkBack creates the environment needed for the application to be functional. A screen-reading functionality is available, which acts as a personal assistant by reading aloud all the options available on the screen. It allows for differentiating titles and texts and provides all the information about the functionalities of the buttons. All three available languages are supported.

### 3.2. The App’s Core Modules

#### 3.2.1. Pose Estimation Mechanism

**Figure 3:**
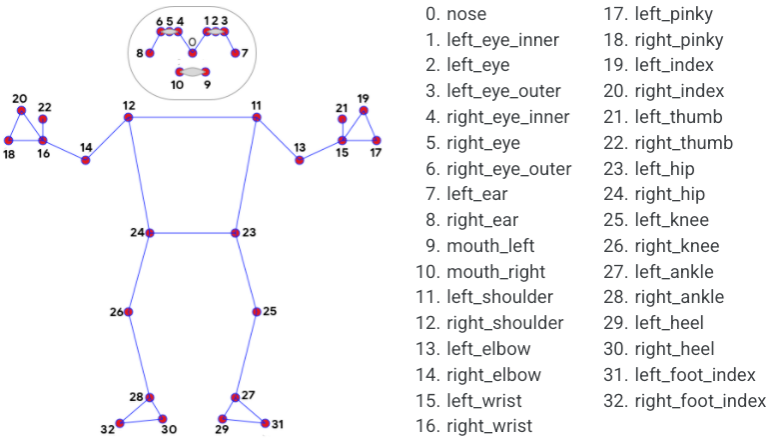
MediaPipe’s 33 key points [29].

The core of the system relies on a pose estimation mechanism, capturing and analyzing the patient’s movements when performing exercises using the smartphone’s camera and the device’s processing capabilities. The Cartesian coordinates of 33 key points on the patient’s body (Figure 3) are extracted using the BlazePose [28] function within the MediaPipe [29] framework. This toolkit enables retrieving detailed information on the face and hands of the human body, important for implementing rehabilitation exercises. Its low processing requirements also make it perfect for mobile devices.

Two different approaches are available for analyzing the patient’s exercises: real time and offline.

The first involves directly capturing and then feeding images using the mobile device’s camera when carrying out the exercise. To do this, the patient must select the desired exercise from the given list in the main menu and, after pressing the record button, the system simultaneously records the video and feeds the frames to the key-point detector. Since this tool does not have the capacity to analyze all the frames captured by the camera, only a selected number of frames is considered.

The second involves analyzing videos from the image gallery on the patient’s device. Upon choosing a pre-recorded video, a set of frames is extracted using the FFmpeg [30] tool and saved in the device’s cache before being fed to the key-point detector.

**Figure 4:**
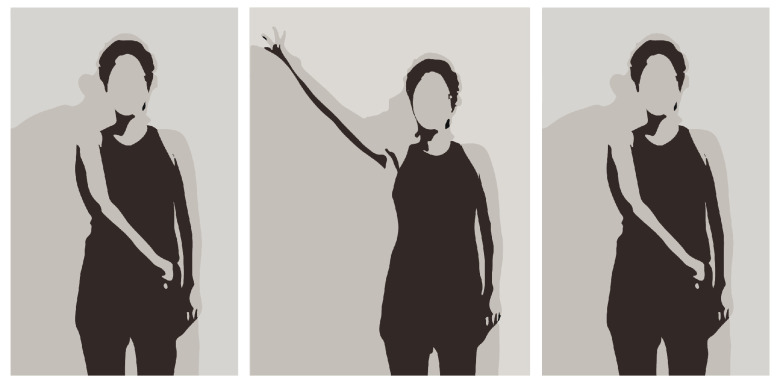
Frames of recorded video of a participant performing the diagonal arm exercise.

**Figure 5:**
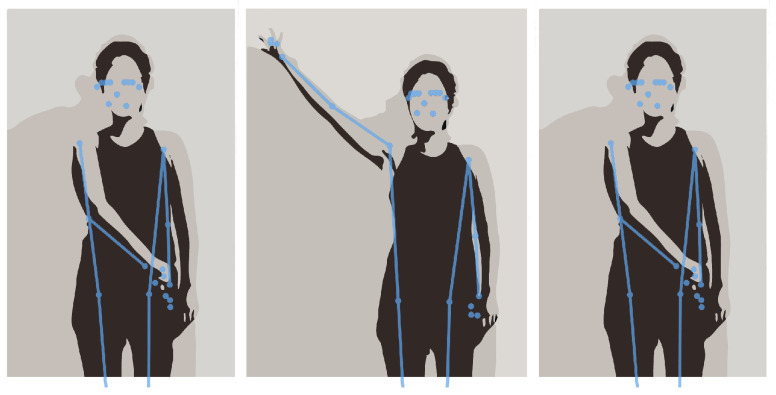
Pose drawing of performing the diagonal arm exercise.

The gathered data can then be used for two distinct purposes: pose drawing and analysis of the exercise. The pose drawing feature allows the patient to see a simple drawing of his/her pose on screen. This is only available while the user is recording the exercise and can be turned on or off depending on the user’s preferences. The pose is redrawn every time the system finishes processing another frame. Figure 4 and Figure 5 illustrate the pose-drawing feature.

#### 3.2.2. Exercise Analysis

One of the most important functionalities of the proposed solution is the automatic analysis of the quality of the performed exercise. To create the ground truth, reference videos were provided by professional physiotherapists and compared with the videos captured by the patient during his/her sessions. To reduce the processing time and the computational resources needed, the reference videos were previously analyzed, and the relevant information was extracted and stored as a Json file in the device’s memory. Two distinct methods were implemented in order to compare the ground truth with the patient’s execution of the exercise: cosine similarity and dynamic time warping (DTW).

##### Cosine Similarity

The similarity of the cosines can be obtained using the following formula:

(1) $$Sim_{cos(\vec{x},\vec{y})}=\frac{\vec{x}\cdot \vec{y}}{\left|\right|\vec{x}\left|\right|\cdot \left|\right|\vec{y}\left|\right|}$$

This metric enables a comparison of the angle between two vectors, returning a similarity value in the range of [−1, 1], which is usually reconverted to the range of [0, 1], refraining from negative numbers. For our analysis, 33 comparisons were made using cosine similarity—one between each key point of the model video and the patient video, i.e., we formed a three-dimensional vector with the Cartesian coordinates of each key point and compared these two vectors.

After comparing the 33 key points, the average similarity of the frame was calculated, and if this value was greater than a predefined limit (in our case, a value greater than 90%), that frame was defined as “correct”. After analyzing the entire video, using the total number of “correct” frames and the total number of frames, it was possible to classify the quality of the execution of the exercise.

##### Dynamic Time Warping

The cosine similarity approach does not take into account the fact that a patient may execute the exercise at a different speed than the reference one. To overcome this limitation, a DTW algorithm was implemented, which calculates the similarity between two sequences that can vary in time or space. In other words, this algorithm calculates the similarity between two time series with different durations or time-lagged actions.

The implementation of this algorithm increases robustness against numerous variables that can be introduced into the system, such as a patient starting an exercise after the start of the recording, a patient performing an exercise at a different speed, or even considering videos with different frame rates.

DTW was applied to compare the key-point sequences derived from the video data. By focusing on subsets representing the head, trunk, and shoulder, DTW facilitated a fine-grained evaluation of movement patterns. The implementation utilized the FastDTW [31] algorithm for efficient computation without compromising accuracy. The DTW method calculates a distance metric that inherently spans a wide range of values. To facilitate a more intuitive comparison with other methods, we transformed the DTW distances into z-scores, which represent the number of standard deviations a particular distance is from the mean. The transformation ensures that the DTW values are comparable across different exercises and methods.

A z-score of 0 corresponds to a distance equal to the mean, and a z-score of 1 indicates a distance of 1 standard deviation above the mean. Consequently, we observed z-scores of 0 for cases where the DTW distance was equal to the mean and z-scores of 1 for the maximum DTW distance. These extreme values, 0 and 1, were then converted to percentages for ease of interpretation, where 0% represents the mean DTW distance, and 100% represents the maximum DTW distance. The 0% and 100% values in the DTW method were transformed to make them comparable and interpretable within the context of this analysis, aligning them with the percentile distribution of DTW distances across all exercises and methods.

## 4. Experimental Setup

In order to verify the effectiveness of the pose detection mechanism and the methods for evaluating the proposed solution, we conducted a clinical study. This study involved 15 participants, who included employees from the Center for Rehabilitation Research. The participants were selected based on specific criteria, excluding those with musculoskeletal and neurological conditions that could influence exercise performance, a history of persistent pain associated with the shoulder complex, and extreme obesity (BMI greater than 40 kg/m^2^). The study design was cross-sectional, and various tools were used for data collection and participant characterization. Further details about the study design and participant selection can be found in our previous work [1].

For this clinical study, we selected QTM (Qualisys Motion Capture System, Qualisys AB, Göteborg, Sweden), an optoelectronic system renowned for its precision in capturing and analyzing 3D motion data. This system was used to assess the joint positions of the shoulder, elbow, wrist, head segment, and trunk of our 15 participants during their rehabilitation exercises. The participants were marked using QTM markers, and their movements were recorded in a controlled environment to ensure the accuracy of the data collected. The videos were recorded using an iPhone 14 (Apple Inc., Cupertino, CA, USA) camera placed 2 m away from the users. The camera was oriented horizontally, capturing the frontal view of the participants’ bodies. The participants were instructed to perform the exercises facing the camera.

**Figure 6:**
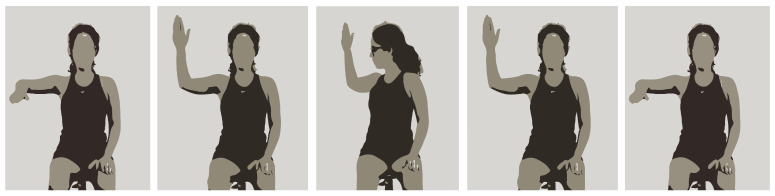
Illustration of the steps required to perform the seated exercise.

Two different shoulder rehabilitation exercises were conducted in this controlled environment. The first corresponded to the movement of the arm according to the exercise in Figure 6. For this exercise, the participant was instructed to start the exercise seated with their arm in a 90° position in relation to their trunk and their elbow in a 90° position in relation to their arm. After this, the participant was instructed to fully externally rotate their shoulder and trunk to the same side of the arm as far as possible, holding this position for 3 s before returning to the starting position.

The second corresponded to the movement of the arm when the participant was standing. The first stage of this exercise consisted of keeping the hand closed (punch position) and the shoulder internally rotated with the elbow extended on the side of the hip from the opposite side to the shoulder on which the exercise was being performed. After this, the participant was instructed to gradually open their hand while performing shoulder external rotation and elevation (simultaneously in frontal and sagittal planes), until it was at a 45° angle to their head. To finish, the participant was instructed to make the opposite movement to return to the starting position. This exercise is illustrated in Figure 5.

To better observe the strengths and weaknesses of the developed application, the exercise evaluation was separated into three distinct parts: head, trunk, and shoulder. By segmenting the evaluation into these three distinct parts, a holistic view of the system’s capabilities and limitations was attained. This multifaceted approach not only provided a thorough understanding of the system’s functionality but also guided the implementation of targeted enhancements and optimizations.

Concurrently, the participants’ movements were also recorded using the developed smartphone application. This dual recording approach allowed us to process and compare the data using both the DTW and cosine similarity methods. To analyze movement variation with QTM, angles were calculated for each segment between two lines formed by anatomical markers. This provided us with a detailed understanding of each participant’s movement patterns and allowed us to accurately assess their performance during the exercises.

QTM provided high-precision tracking of the reflective markers placed on the participants, enabling the capturing of intricate details of their movements. We leveraged QTM to obtain ground-truth data for specific exercises, fostering a comparative analysis between video-derived key points and marker-based motion capture. DTW was applied to compare the key-point sequences derived from the video data, and cosine similarity was employed to assess the similarity of the movement patterns. This method complemented the temporal analysis of DTW and the marker-based precision of QTM. By calculating the cosine of the angle between key-point vectors, we gained additional insights into the consistency of movements across different exercises.

The integration of these three methods allowed for a multi-faceted examination of participant performance. DTW captured temporal dissimilarity, QTM provided a benchmark for accuracy, and cosine similarity measured pattern resemblance. This triad of methodologies allowed us to discern subtle variations and draw comprehensive conclusions about the effectiveness of rehabilitation exercises.

## 5. Results

Considering the above, we present a comparative analysis of the three methods used for our exercises: QTM, the dynamic time warping (identified as “DTW”) method, and the cosine similarity method (identified as “APP”).

**Figure 7:**
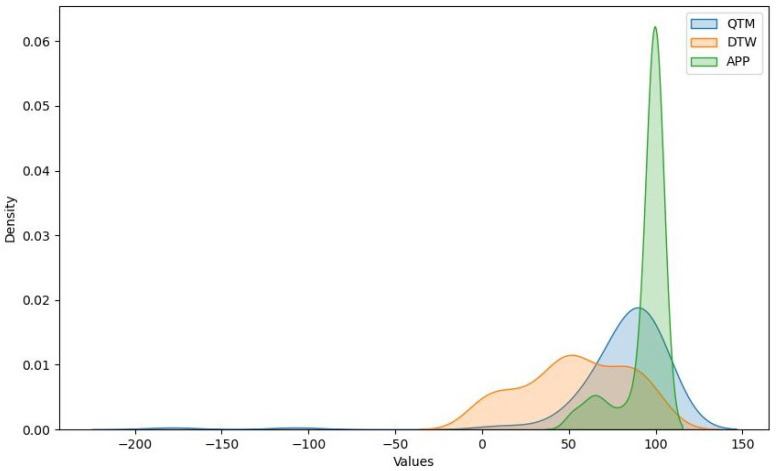
Density plot for QTM, DTW, and cosine methods.

**Figure 8:**
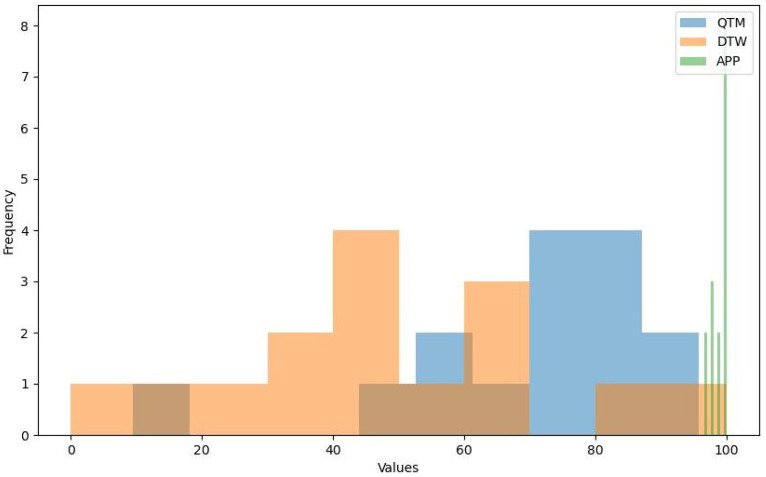
Histogram of HEAD_DIAG exercise.

**Figure 9:**
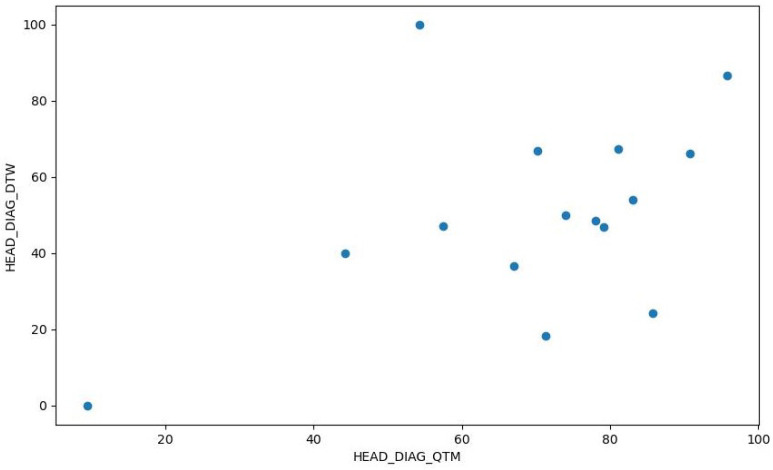
Scatter plot of HEAD_DIAG_QTM vs. HEAD_DIAG_DTW.

**Figure 10:**
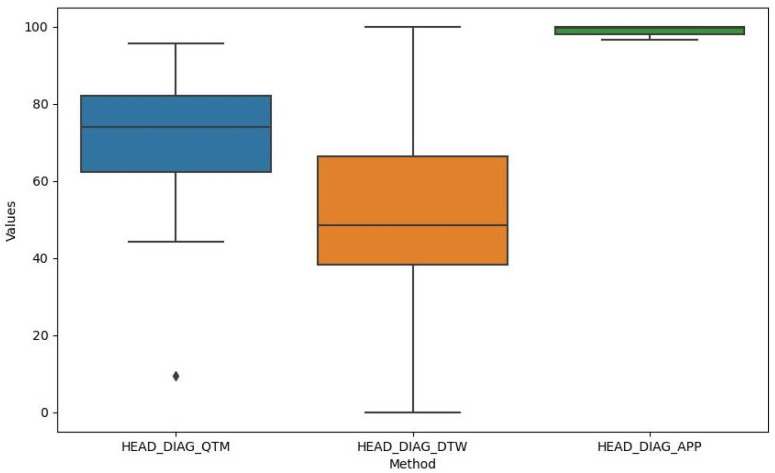
Box plot of HEAD_DIAG exercise.

The data consist of measurements from six different exercises: HEAD_DIAG, TRUNK_DIAG, SHOULDER_DIAG, HEAD_ROT, TRUNK_ROT, and SHOULDER_ROT. These represent the measures of the diagonal exercises of the head, trunk, and shoulder and the rotational exercises of the head, trunk, and shoulder, respectively. For each exercise, we obtained measurements using the three methods. A density plot containing these measurements is presented in Figure 7. Figure 8 shows a histogram of the distribution of the values for the HEAD_DIAG exercise across the three methods. Figure 9 displays a scatter plot that visualizes the relationship between the QTM and DTW methods on the diagonal head exercise. Finally, Figure 10 shows a box plot with the values for the diagonal head exercise across the three methods.

The QTM method produced a wide range of values, with a mean of 78.8 and a standard deviation of 21.61. The minimum and maximum values were 9.47 and 95.77, respectively. QTM exhibited negative skewness for all exercises, indicating that the distribution of the values was skewed to the right, with more values falling above the mean. The kurtosis was positive for HEAD_DIAG_QTM and SHOULDER_DIAG_QTM, indicating a leptokurtic distribution with heavy tails and a sharp peak, suggesting potential outliers. The right-skewed distribution observed in the QTM method suggests that this method often produced values above the mean. This could indicate a higher sensitivity to variations in the performance of shoulder rehabilitation exercises, making it potentially more suitable for detecting subtle improvements or deteriorations in patient performance over time.

After transformation into z-scores and then into percentages (as mentioned previously), the DTW method exhibited a mean of 53.74 and a standard deviation of 25.59. The minimum value of 0% represents the mean DTW distance, whereas the maximum value of 100% represents the maximum DTW distance. DTW exhibited near-zero skewness for HEAD_DIAG_DTW, TRUNK_DIAG_DTW, and SHOULDER_DIAG_DTW, indicating a symmetrical distribution around the mean. The kurtosis was also near zero for these exercises, suggesting a mesokurtic distribution similar to a normal distribution. The near-zero skewness and kurtosis for the diagonal exercises in the DTW method indicate a symmetrical and normal-like distribution around the mean. This suggests that the DTW method can provide a balanced measure of performance that is not overly influenced by extreme values. Therefore, it could be a reliable choice for a general evaluation where extreme performance is not the primary concern.

The APP method exhibited a mean of 94.08 and a standard deviation of 1.18, indicating that the values were closely clustered around the mean. However, there was some variation in the data, as indicated by the minimum value of 51.67 for SHOULDER_ROT_APP. The APP method exhibited negative skewness for HEAD_DIAG_APP and HEAD_ROT_APP and positive skewness for SHOULDER_ROT_APP, indicating that the distribution of values was skewed to the right for the former two and the left for the latter. The kurtosis was negative for HEAD_DIAG_APP and HEAD_ROT_APP, indicating a platykurtic distribution with light tails and a flat peak, suggesting fewer outliers. The negative skewness and kurtosis for the head exercises in the cosine similarity method indicate a platykurtic and left-skewed distribution with fewer outliers. This could mean that this method is less sensitive to variations in performance, making it potentially more suitable for exercises where consistency is more important than peak performance.

We conducted a one-way ANOVA to compare the effect of different similarity metrics on exercise performance. This test was chosen because we were comparing the means of more than two groups (the different similarity metrics) based on one dependent variable (exercise performance). The ANOVA results, presented in Table 1, provide insights into the differences between the three methods across all exercises. Overall, all results had a *p*-value lower than 0.05, indicating a statistically significant difference between the means of the three methods for all exercises.

Following the one-way ANOVA, we conducted post hoc comparisons using Tukey’s Honestly Significant Difference (HSD) test to determine which specific groups (similarity metrics, in this case) differed significantly from each other.

The results of Tukey’s HSD test, presented in Table 2, indicate that for most exercises and pairs of methods, there was a significant difference, with *p*-values less than 0.05. This suggests that the choice of similarity metric significantly impacted the outcome of the exercises. However, there were a few exceptions where no significant difference was observed:

- For SHOULDER_DIAG, there was no significant difference between APP and QTM.
- For HEAD_ROT, there was no significant difference between APP and DTW and between DTW and QTM.
- For TRUNK_ROT, there was no significant difference between APP and QTM.
- For SHOULDER_ROT, there was no significant difference between APP and DTW and between APP and QTM.

The statistically significant differences observed between these methods across all exercises underscore the importance of selecting an appropriate method for specific exercises. Depending on whether we want to detect subtle changes, balance extreme performance, or emphasize consistency, one method may be more appropriate than others. These findings highlight the need for practitioners to carefully consider their choice of method when evaluating patient performance in shoulder rehabilitation exercises.

The results from the one-way ANOVA and the subsequent Tukey’s HSD test suggest that there were statistically significant differences in measurements between the QTM, DTW, and APP methods for most exercises. However, the specific pairs of methods that differed significantly varied depending on the exercise.

## 6. Discussion

Our study aimed to develop a novel smartphone-based approach to shoulder rehabilitation using pose estimation and exercise evaluation methods. We conducted a clinical study with 15 participants and compared our methods with QTM, an optoelectronic system for motion capture. Our results showed that our methods were able to measure the correctness of rehabilitation exercises with reasonable accuracy and reliability and provide feedback to patients and therapists.

We conducted a comparative analysis of three methods to validate the statistical significance of our results. The analysis of the QTM, DTW, and cosine similarity methods revealed distinct characteristics and performance across different shoulder rehabilitation exercises. The QTM method demonstrated a right-skewed distribution, suggesting that it often produced values above the mean. In contrast, the DTW method exhibited a symmetrical and normal-like distribution for diagonal exercises. The cosine similarity method showed a platykurtic and left-skewed distribution for head exercises, indicating fewer outliers. Statistical tests further highlighted significant differences in measurements between these methods across all exercises.

These findings underscore the importance of selecting an appropriate method for specific exercises to ensure accurate and reliable performance evaluations in (shoulder) rehabilitation. Furthermore, the relatively high degree of accuracy achieved by our pose estimation algorithm means that patients can rely on our system to perform their rehabilitation exercises correctly. This could lead to more effective rehabilitation sessions and better patient outcomes. Also, these findings align closely with the goals and objectives of our study. We set out to develop a more accessible and effective solution for shoulder rehabilitation, and our findings demonstrate that we have achieved this goal. The use of a smartphone-based system makes our solution widely accessible, and the high accuracy of our pose estimation algorithm ensures that it is effective.

Our results contribute to the field of mobile health and rehabilitation by demonstrating the feasibility and effectiveness of using smartphone cameras and pose estimation algorithms to support shoulder rehabilitation exercises, thereby aiding in their at-home monitoring. Our approach offers several advantages over existing solutions, such as wearable devices or camera-based systems, including lower costs, greater convenience, greater accessibility, and a better user experience. It also complements traditional face-to-face sessions with therapists by enabling autonomous and remote sessions with real-time feedback and monitoring.

The potential clinical implications of our findings are significant. Our smartphone-based approach could broaden access to physical therapy, particularly for patients who may have limited access to in-person therapy sessions. By enabling patients to perform exercises at home with real-time feedback, we could see increased adherence to therapy regimens and potentially faster recovery times.

However, our study has some limitations that should be considered when interpreting our results. The small sample size and the use of volunteers may limit the generalizability of our findings. While our findings are promising, we believe that different patient populations may present unique challenges and needs. For instance, patients with different types of shoulder injuries or varying degrees of mobility may require specific, custom-made approaches. Furthermore, factors such as age, tech literacy, and/or access to a compatible smartphone could also influence the effectiveness and usability of our system. Therefore, we want to make it clear that there is a need for further research and user testing across diverse settings to fully understand the proposed system’s generalizability.

Another limitation of our study is that we only tested our method on frontal videos of the participants, which may not reflect the variability of real-world scenarios. Although MediaPipe claims to support different camera angles and viewpoints, we have not verified the accuracy and stability of our method for other views, such as lateral or oblique. We also only tested two types of shoulder rehabilitation exercises, which may not cover the full range of exercises that are prescribed by therapists or performed by patients. Furthermore, our study did not assess the long-term effects or outcomes of using our smartphone-based approach on the patients’ recovery processes or quality of life.

In conclusion, we believe that our research could have a profound impact on the field of shoulder rehabilitation. The use of a pose estimation algorithm provides an objective, quantifiable measure of a patient’s progress. This data-driven approach could enable physical therapists to more effectively tailor treatment plans to each patient’s unique needs and progress, potentially improving treatment outcomes.

## 7. Conclusions

This paper presents a novel smartphone-based approach for shoulder rehabilitation. Our approach leverages pose estimation and exercise evaluation methods to provide real-time feedback to patients and therapists. The results from our clinical study with 15 participants demonstrate the effectiveness and reliability of our methods in measuring the correctness of rehabilitation exercises.

Our findings contribute significantly to the field of mobile health and rehabilitation. The advantages of our approach over existing solutions, such as wearable devices or camera-based systems, include lower costs, greater convenience, greater accessibility, and a better user experience. Furthermore, our approach complements traditional face-to-face sessions with therapists by enabling autonomous and remote sessions.

The comparison of our methods with QTM provided valuable insights into the strengths and weaknesses of different methods for evaluating shoulder rehabilitation exercises. This comparison highlighted the importance of selecting an appropriate method for specific exercises.

In our discussion, we identified and explored certain limitations inherent in our research. Future studies will address these limitations by conducting larger-scale and longer-term investigations with more diverse and representative samples. More types of exercises will be tested, and the impact of our approach on clinical and patient-reported outcomes will be measured. Also, the app will be evaluated on different views and compared to the results using the frontal view. This will provide more insights into the robustness and applicability of our method for remote monitoring and rehabilitation.

In summary, we propose a promising approach for shoulder rehabilitation that harnesses the power of mobile technology. While further research is needed to fully realize its potential, our findings provide a strong foundation for future work in this exciting field.

## Figures and Tables

**Table 1:** Results of one-way ANOVA.

Exercise	F-Value	*p*-Value
HEAD_DIAG	24.11	<0.001 *
TRUNK_DIAG	35.30	<0.001 *
SHOULDER_DIAG	53.76	<0.001 *
HEAD_ROT	3.79	0.03 *
TRUNK_ROT	14.38	<0.001 *
SHOULDER_ROT	9.22	<0.001 *

**Table 2:** Results of Tukey’s HSD test.

Exercise	Group 1	Group 2	Mean Difference	*p*-Value
HEAD_DIAG	APP	DTW	−48.7122	<0.001 *
HEAD_DIAG	APP	QTM	−29.4727	<0.001 *
HEAD_DIAG	DTW	QTM	19.2395	0.0249
TRUNK_DIAG	APP	DTW	−49.271	<0.001 *
TRUNK_DIAG	APP	QTM	−19.2553	0.0062 *
TRUNK_DIAG	DTW	QTM	30.0157	<0.001 *
SHOULDER_DIAG	APP	DTW	−53.9211	<0.001 *
SHOULDER_DIAG	APP	QTM	−3.3887	0.8306
SHOULDER_DIAG	DTW	QTM	50.5324	<0.001 *
HEAD_ROT	APP	DTW	−35.0981	0.1556
HEAD_ROT	APP	QTM	−49.8967	0.0277 *
HEAD_ROT	DTW	QTM	−14.7985	0.7083
TRUNK_ROT	APP	DTW	−37.157	<0.001 *
TRUNK_ROT	APP	QTM	−5.862	0.7131
TRUNK_ROT	DTW	QTM	31.295	<0.001 *
SHOULDER_ROT	APP	DTW	−17.8507	0.0744
SHOULDER_ROT	APP	QTM	16.2073	0.1145
SHOULDER_ROT	DTW	QTM	34.058	<0.001 *

## Data Availability

Data are contained within the article.
